# Supplementary material for: Balance recovery schemes following mediolateral gyroscopic moment perturbations during walking
Source: PLoS One. 2024 Dec 31;19(12):e0315414. doi: 10.1371/journal.pone.0315414 (PMC11687818; doi:10.1371/journal.pone.0315414)
Supplement: Attachment — Submitted filename: Rebuttal_Letter .pdf [file pone.0315414.s001.pdf]

Authors' Response to Reviewers' Comments on Submission PONE-D-24-24945

Balance Recovery Schemes for Mediolateral Gyroscopic Perturbations During Walking

Dear **Prof. Mengarelli**,

We would like to express our gratitude to you and the reviewers for your time and constructive feedback, which has significantly improved our manuscript. We carefully considered all comments and revised the paper accordingly. To address the comments, suggestions, and concerns, we modified the paper and added new explanations/clarifications.

Editor's comment

Reviewers were overall positive about the manuscript, and although no significant technical issues have been recognized, I would suggest to revise the paper taking into account carefully the Reviewers comments and suggestions, since some concerns have been raised in particular regarding the added value of this work to existing literature, and the general impact.

Thank you for the positive evaluation of our manuscript. We have revised the paper to address the comments and suggestions raised by the reviewers.
The main constructive changes in the revised version are as follows:

1. The limitations of previous perturbation devices are discussed, and the innovative aspects of this work are clarified.
2. The derivative of whole-body angular momentum with respect to time has been added to the Results section.
3. The influence of the angular momentum perturbator (AMP) device on nominal gait is addressed in the Discussion section.

All the changes to the main article are highlighted in yellow in this letter and in the revised manuscript (as suggested by the editor).

In the following, a detailed response to the comments is given. The comments and our replies are in ***bold italic*** and normal faces, respectively.

Responses to Reviewer #1:

This paper investigates how human test participants respond to pure WBAM perturbations in two directions and at two moments during the human gait cycle. The paper is very well written and nicely to the point.

R1-1: The only feedback I have is rather minor:

L47: you contrast your mobile/portable AMP system perturbations with "traditional force-based and whole-body pitch angle perturbations". I understand your point of mobility versus statically connected to the treadmill(-base), now allowing you to do overground walking etc.. However, it is not really clear what you really mean with limitations of "force-based and whole-body pitch angle perturbations". Is it not possible to purely perturb AM with those systems? I think you could be a bit more explicit here.

✓ **Response:** Thank you for the positive feedback and valuable suggestions that helped improve our work. We have carefully addressed each of the comments and revised the paper accordingly, as detailed below.

Conventional methods for applying perturbations—such as perturbations induced by slipping or tripping [5,6], treadmill accelerations and decelerations [7], external forces applied to the pelvis or shoulder via pushes or pulls [8-10], or swing leg obstacle collisions [11]—result in changes to both linear and angular momentum. These perturbations affect the translational motion of the center of mass and indirectly disturb angular momentum, requiring complex corrective actions that engage various response mechanisms. As a result, isolating specific strategies for regulating whole-body angular momentum becomes challenging. In contrast, whole-body pitch angle perturbations, achieved by applying two simultaneous perturbations of equal magnitude in opposite directions [12], allow for angular momentum modulation without affecting linear momentum. However, such devices are typically not portable and require mechanical linkages between the body and external structures, which impose physical constraints and limit the movement of biological joints. Additionally, these devices are restricted to perturbations in the sagittal plane, necessitating substantial mechanical modifications for perturbations in other planes.

The AMP system utilized in our study addresses these limitations by specifically targeting angular momentum regulation during walking, particularly in the torso segment. This capability allows for the independent modulation of angular momentum, enabling a focused study on the rotational components of human balance.

We agree with the reviewer that the limitations associated with the conventional perturbation devices may not have been clearly explained. Therefore, in our revised version, we have included a paragraph in the introduction section to clarify these limitations.

✓ **Action:**

Page 2, 'Introduction' Section:

Prior research has highlighted the complex strategies individuals use to maintain balance in a variety of destabilizing scenarios [1–4]. Most studies, however, have concentrated on perturbations caused by slipping or tripping [5,6], treadmill accelerations and decelerations [7], external forces applied to the pelvis or shoulder via pushes or pulls [8-10], or swing leg obstacle collisions [11]. These perturbations affect both linear and angular momentum, requiring complex corrective actions across multiple response mechanisms, which complicates the isolation of specific strategies for regulating WBAM. In contrast, whole-body pitch angle perturbations, achieved by applying two simultaneous perturbations of equal magnitude in opposite directions [12], allow for angular momentum modulation without affecting linear momentum. However, such devices are typically not portable and require mechanical linkages between the body and external structures, which impose physical constraints and could limit the movement of biological joints. Additionally, these devices are restricted to perturbations in the sagittal plane, necessitating substantial mechanical modifications for perturbations in other planes.

R1-2: Fig 1: I find Fig 1.c to be not so clear in what it is trying to show, since I cannot really see what foot is in the air or on the ground. Possibly both a front/side view might make it fully unambiguous. Another very minor remark: in the a) is a human, b) is a mannequin and c) is a skeleton. Possibly some consistency might be preferred (human silhouettes?).

✓ **Response:**

We appreciate the reviewer's attention to detail. In response to your comment, we have replaced the mannequin with human silhouettes. Additionally, in Fig1.c, we have included ground symbols beneath the feet to clearly indicate which foot is in contact with the ground.

✓ **Action:**

Please refer to the end of this document, or the revised version of the manuscript, to view the updated figure.

R1-3: Fig 2.:

A- Maybe it is image compression imposed by the PLOS one reviewing system, but the figures are VERY compressed, almost to the extent that the text and lines in plots becomes very difficult to read (blurry) and distinguish: I cannot see/distinguish the H_CTRL and theta_CTRL lines.

B- "across all subjects and trials", so we are looking at the mean? Please make a bit more explicit what we are really looking at. Why is the perturbation torque not trapezoidal (control bandwidth limitation? consequence of averaging?)

C- If possible, also show (approximate) derivative of the WBAM, i.e. dH/dt, and perturbation torque together in 1 plot, (and possibly their estimated difference) because then we can infer the net ML CoM Torque that the participant generates to regulate the WBAM back to nominal behavior. Because afterwards, one can start thinking about how this feedback moment will be distributed over the joints, which will inform us more mathematically about relative weighting the participants solve this redundancy given the foothold constraints and future planning (leading to hip strategy).

✓ **Response:**

A- The quality issue you observed is due to the figure file requirements imposed during submission. We originally generated all figures as vector files to ensure high resolution. In our resubmission, we have uploaded the figures separately in PDF format, along with the revised manuscript, to enhance clarity. For your convenience, we have also included all figures at the end of this document for your review.

B1- Thank you for your comment. As you stated, the graphs plotted in figure 2 are grand means and we agree that this might not have been clearly mentioned. In the revised version, we have explicitly mentioned this in the caption of the figure.

B2- The desired AMP output torque was designed to follow a symmetric trapezoidal profile, which accounts for the gimbal motor's limited capacity to accelerate or decelerate the gimbal structure and generate the required gyroscopic effect [13]. In this work, a gimbal motor torque of approximately 12 Nm was needed to produce the desired perturbations. Due to the motor dynamics and the influence of the low-level feedback loop controlling the gimbal motor's reference torque [14], the actual output torque exhibits a curved trapezoidal shape. The figure below shows the AMP torque profiles generated for a single subject across different trials. Averaging the AMP torque across all subjects and trials would naturally smooth out the trapezoidal shape further. In the revised manuscript, we have clarified the reason behind the smooth shape of the AMP torque.

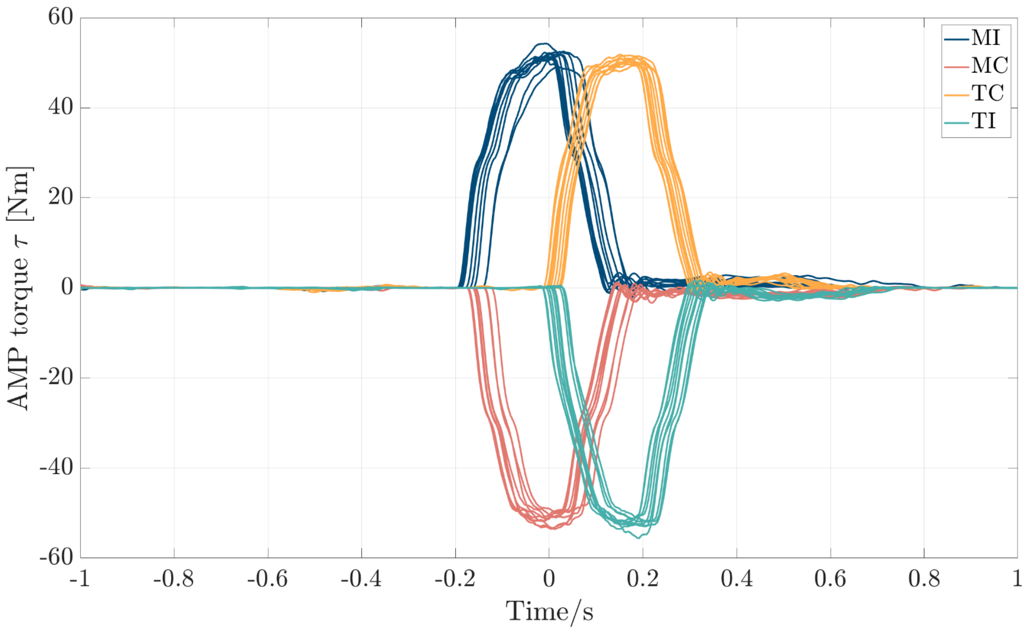

C- In response to your suggestion, we have revised Fig. 2 in the manuscript (see the updated version at the end of this document). The new version now includes the derivative of WBAM, and we have aligned the plots vertically for easier comparison. We have also added standard deviations to the graphs. To maintain readability, we opted not to overlay the perturbation profiles with other quantities, such as trunk angle and WBAM, on the same plot. Additionally, we have updated the corresponding section in the Results to reflect these changes.

✓ **<u>Action:</u>**

Page 5, Figure 2:

Perturbation profiles and their effect. Grand mean ± standard deviation of the perturbation profiles and their resulting impact on trunk angle (θ), whole-body angular momentum (WBAM, *H*), and the time derivative of WBAM (*dH/dt*) in the frontal plane, averaged across all subjects and trials. In all figures, the time point 0 indicates the moment when the left leg comes into contact with the second force plate. Horizontal lines overlaid on the graphs indicate regions where signals in the perturbed cases exhibit significant differences compared to control trials ($p < 0.05$)...

Page 3, 'Perturbation Device' Section:

This profile accounts for the gimbal motor's limited ability to accelerate or decelerate the gimbal structure and generate the desired gyroscopic effect [13]. In this study, a gimbal motor torque of approximately 12 Nm was required to produce the desired perturbations.

Page 5, 'Perturbation profile and effect' Section:

The AMP perturbations were designed to adopt a symmetric trapezoidal profile. However, due to the motor dynamics and the influence of the low-level feedback loop controlling the gimbal motor's reference torque [14], the actual output torque exhibited a curved trapezoidal shape.

Page 6, 'Perturbation profile and effect' Section:

Due to the perturbations induced, the trunk angle displayed ML deflection peaks of +6.9 deg to +10.0 deg for rightward (*MI* and *TC*) perturbations, and peaks ranging from −11.7

deg to −13.4 deg for leftward (*MC* and *TI*) perturbations, relative to the trunk posture observed in control trials (*CTRL*) without perturbations.

Regarding whole-body angular momentum (WBAM) in the frontal plane, the AMP perturbations significantly elevated WBAM peaks in alignment with the direction of perturbation. For rightward perturbations (*MI* and *TC*), WBAM peaks increased to between +0.11 and +0.15. Conversely, leftward perturbations (*MC* and *TI*) led to initial WBAM peaks ranging from −0.11 to −0.12.

The net ML torque, derived as the first derivative of whole-body angular momentum, revealed that immediately after the perturbation, the net torque spiked due to the sudden change in angular momentum caused by the external torque. For rightward perturbations, dH/dt initially peaked up to +0.73 s^{-1} and +1.07 s^{-1}, while for leftward perturbations, initial peaks ranged from −0.93 s^{-1} and −0.98 s^{-1}. As the body responded, the rate of change of angular momentum reversed sign as participants attempted to apply counteracting torques and regain control, with peaks of up to −1.02 s^{-1} and −1.46 s^{-1} for rightward perturbations and peaks of up to +0.98 s^{-1} and +1.20 s^{-1} for leftward perturbations. Eventually, *dH/dt* approached zero as the participants recovered from the perturbation.

R1-4: Suggestion:

L241: a larger step width -> a wider step

L13,L56,L292 healthy -> able-bodied

✓ <u>**Response:**</u>

We have made revisions to the manuscript following the reviewer's recommendations.

R1-5: Finally:

Do whatever you wish with the following piece of trivia (possibly ignore), but it could make Eq (1) shorter by 1 symbol. In Eq (1) there is no need to do "-v_body^COM". Those mass scaled contributions of the whole body com velocity all cancel out in the total sum. Consider 2 point masses

(m1,m2) that have no inertia (tensor) that have their own com at r1, r2 and a barycenter (whole body com) at rc, with velocity vc (expressed in some frame). I use parameter A to add (A=1) or remove (A=0) the vc contribution. If one can show that the WBAM is independent of A, the point is made. Proof by MATLAB symbolic example:

```
syms m1 m2 A
r1 = sym('r1',[3 1]);r2 = sym('r2',[3 1]);
v1 = sym('v1',[3 1]);v2 = sym('v2',[3 1]);
rc = (r1*m1+r2*m2)/(m1+m2);vc = (v1*m1+v2*m2)/(m1+m2);
contribution1 = (cross(r1-rc,m1*(v1-A*vc)))
contribution2 = (cross(r2-rc,m2*(v2-A*vc)))
WBAM = simplify(contribution1+contribution2) %cancellations happen here
```

Which shows that WBAM is independent of A and vc can be omitted. This trivially generalizes to systems of N particles and also holds when product I*w is reintroduced for rigid bodies.

✓ **Response:**

This is an interesting observation. Accordingly, we have removed the term v_{body}^{CoM} from the formula in the revised manuscript.

✓ **Action:**

Page 4, 'Outcome Measures' Section:

$$H = \sum_{i=1}^{n}\left[\left(r_i^{CoM} - r_{body}^{CoM}\right) \times m_i v_i^{CoM} + I_i \omega_i\right]$$

Responses to Reviewer #2:

In this paper the authors reported on findings related to using a "backpack"-esq perturbation device which applies an angular momentum-based perturbation that can be used during overground walking. In this study the authors performed frontal plane perturbations.

In general the paper was well written. It appeared to be technically sound and rigorous.

R2-1: ***In spite of being well written, I am left wondering about the "why" for this paper. On the one hand, I appreciate the effort to develop a perturbation device for overground walking. On the other hand, we already know a good deal about perturbation responses in healthy adults. The authors allude to implications for interventions for elderly individuals in their conclusion, however, I fail to see the direct relationship. We know from other populations that balance responses are not equivalent to healthy adults, so it would be reasonable to suspect that the strategies may not carry over to those elderly needing fall prevention interventions. So, I think the authors need to carefully reconsider the conclusions and implications of the present study after considering that other populations may not employ the same strategy as young healthy adults.***

✓ **Response:**

Thank you for your positive feedback on our work and for your valuable suggestions. We have carefully considered your comments and refined our work accordingly.

A) As the reviewer correctly pointed out, prior research has shed light on the strategies individuals use to maintain balance when responding to perturbations and probe the relationship between balance and recovery strategies. However, many of these studies have primarily focused on perturbations induced by slipping/tripping over obstacles or external forces applied at the pelvis or shoulder, which challenge the center of mass's translational motion and indirectly affect angular momentum. These perturbations require intricate corrective actions that may involve multiple response mechanisms. This complexity makes it difficult to isolate specific strategies for regulating whole-body angular momentum.

Our AMP device offers a key advantage over other perturbation devices by specifically targeting the regulation of angular momentum during walking, allowing for isolated investigation of human motor control in response to targeted perturbations. This feature allows us to distinguish

between the modulation of linear and angular momentum, a distinction that has often been overlooked in prior studies. Additionally, AMP's portability eliminates the need for mechanical linkages between the body and external structures, minimizing confounding factors related to physical constraints. It also allows for unrestricted movement of biological joints, making it a highly suitable candidate for both perturbation and balance-assistive applications.

Moreover, AMP enables a deeper exploration of the mechanisms underlying balance control in the frontal plane during gait—insights that are challenging to obtain using conventional perturbation methods. <u>To the best of our knowledge, this study is unique in its investigation of angular momentum perturbations in the frontal plane during overground walking with minimal effect on the WBLM, an area that has not been explored before.</u> By addressing this gap, our work contributes valuable insights into the sensorimotor control of balance.

Since these points were not clearly outlined in our previous submission, we have now included them in the introduction section of the revised manuscript.

B) Although balance responses in elderly individuals may differ from those in healthy young adults due to factors like reduced muscle strength, delayed reaction times, and altered neuromuscular control, investigating responses to AMP perturbations in this study can offer valuable insights into *human motor control*, which, despite age-related influences, remains fundamentally similar across age groups. In our original conclusion, we aimed to highlight that the insights gained from able-bodied individuals provide a foundational understanding that could inform the design of assistive devices or interventions for elderly individuals. It's important to note that our AMP, while utilized as a perturbation device in this study, also functions as an assistive device [15].

However, we recognize that directly applying these strategies to the elderly population may not be straightforward, as physiological and biomechanical differences must be carefully considered. In response to your comment, we have revised the manuscript to ensure that our conclusions align solely with the reported outcomes for the able-bodied population, excluding any direct implications for the elderly.

✓ **<u>Action:</u>**

Page 2, 'Introduction' Section:

Prior research has highlighted the complex strategies individuals use to maintain balance in a variety of destabilizing scenarios [1–4]. Most studies, however, have concentrated on perturbations caused by slipping or tripping [5,6], treadmill accelerations and decelerations [7], external forces applied to the pelvis or shoulder via pushes or pulls [8-10], or swing leg obstacle collisions [11]. These perturbations affect both linear and angular momentum, requiring complex corrective actions across multiple response mechanisms, which complicates the isolation of specific strategies for regulating WBAM. In contrast, whole-body pitch angle perturbations, achieved by applying two simultaneous perturbations of equal magnitude in opposite directions [12], allow for angular momentum modulation without affecting linear momentum. However, such devices are typically not portable and require mechanical linkages between the body and external structures, which impose physical constraints and could limit the movement of biological joints. Additionally, these devices are restricted to perturbations in the sagittal plane, necessitating substantial mechanical modifications for perturbations in other planes.

Page 3, 'Introduction' Section:

… To the best of our knowledge, this study is unique in its investigation of angular momentum perturbations in the frontal plane during overground walking with minimal effect on the WBLM, an area that has not been explored before. ...

Page 9, 'Discussion' Section:

These insights hold significant implications for the design and enhancement of controllers for robots and assistive devices, offering valuable considerations for clinical interventions.~~, particularly in the context of elderly individuals.~~

R2-2: While no method of applying perturbations (currently) will result in unperturbed walking being 100% identical to overground walking, I would like to see some discussion about the degree to which walking with this device (unperturbed) correlates with regular, unperturbed overground walking. This is potentially important as 16kg is potentially a substantial load for some participants. In addition to

the mass, it also appears rather "bulky" that may impact normal biomechanics. I could conceive that this would alter normal medio-lateral sway and / or increase the posterior position of the center of mass relative to a stepping foot. Moreover, this could easily result in alterations to steady-state walking momenta. Readers may have concerns about these effects. I understand that it appears that the authors have discussed this in their previous papers, but I, nevertheless, feel that a brief discussion is warranted in the present paper.

✓ **Response:**

We appreciate the reviewer's comment and agree that the effects of the AMP's added mass should be addressed. We have addressed this by adding a new subsection, "Methodological Limitations," in the Discussion section of the revised manuscript.

✓ **Action:**

Page 9, Discussion Section:

Methodological limitations

The AMP used in this study was initially designed to explore gyroscopic actuation principles and was therefore not optimized for weight or clinical applications. Consequently, its mass of 16 kg could impose increased physiological strain on the trunk [16], potentially limiting its viability as an assistive device [17, 18]. Our previous analysis of the AMP's effect on nominal gait revealed that the device induces an average trunk flexion of 7.8 deg compared to normal walking without the AMP [19]. This forward tilt of the trunk could influence hip and ankle moments [20]. Other changes in lower-limb joint kinematics were minimal, with deviations of less than 3 deg compared to nominal gait [19]. Importantly, these alterations in gait do not affect the results of the current study, as the baseline (i.e., *CTRL*) for comparing different experimental conditions was established using the AMP device, rather than normal walking. In future work, to address the unwanted effects of the AMP's mass, we plan to use a newer version of the AMP weighing 4.9 kg [21], which consists of two compact pint-sized CMGs, each weighing only 1.2 kg [22].

References

1. Van Mierlo M, Vlutters M, Van Asseldonk E, Van Der Kooij H. Centre of pressure modulations in double support effectively counteract anteroposterior perturbations during gait. Journal of biomechanics. 2021;126:110637.
2. aleshi N, Brownjohn JM, Lamb SE, Zivanovic S, Williams GK. Vector coding reveals the underlying balance control strategies used by humans during translational perturbation. Scientific Reports. 2022;12(1):21030.
3. Leestma JK, Golyski PR, Smith CR, Sawicki GS, Young AJ. Linking whole-body angular momentum and step placement during perturbed human walking. Journal of Experimental Biology. 2023;226(6):jeb244760.
4. Shokouhi S, Sritharan P, Lee PVS. Recovering whole-body angular momentum and margin of stability after treadmill-induced perturbations during sloped walking in healthy young adults. Scientific Reports. 2024;14(1):4421.
5. Pijnappels M, Bobbert MF, van Die ̈en JH. Contribution of the support limb in control of angular momentum after tripping. Journal of biomechanics. 2004;37(12):1811–1818.
6. Martelli D, Aprigliano F, Tropea P, Pasquini G, Micera S, Monaco V. Stability against backward balance loss: Age-related modifications following slip-like perturbations of multiple amplitudes. Gait & posture. 2017;53:207–214.
7. Sessoms PH, Wyatt M, Grabiner M, Collins JD, Kingsbury T, Thesing N, et al. Method for evoking a trip-like response using a treadmill-based perturbation during locomotion. Journal of biomechanics. 2014;47(1):277–280.
8. Zhu RTL, Lyu PZ, Li S, Tong CY, Ling YT, Ma CZH. How Does Lower Limb Respond to Unexpected Balance Perturbations? New Insights from Synchronized Human Kinetics, Kinematics, Muscle Electromyography (EMG) and Mechanomyography (MMG) Data. Biosensors. <2022;12(6):430.
9. Inacio M, Creath R, Rogers MW. Effects of aging on hip abductor-adductor neuromuscular and mechanical performance during the weight transfer phase of lateral protective stepping. Journal of biomechanics. 2019;82:244–250.
10. Lee YJ, Liang JN, Chen B, Aruin AS. Characteristics of medial-lateral postural control while exposed to the external perturbation in step initiation. Scientific reports. 2019;9(1):16817.
11. Eveld ME, King ST, Vailati LG, Zelik KE, Goldfarb M. On the basis for stumble recovery strategy selection in healthy adults. Journal of biomechanical engineering. 2021;143(7):071003.
12. van Mierlo M, Vlutters M, van Asseldonk E, van der Kooij H. Sagittal-plane balance perturbations during very slow walking: Strategies for recovering linear and angular momentum. Journal of biomechanics. 2023;152:111580.

13. Schumacher C, Berry A, Lemus D, Rode C, Seyfarth A, Vallery H. Biarticular muscles are most responsive to upper-body pitch perturbations in human standing. Scientific reports. 2019 Oct 10;9(1):14492.
14. Lemus D, van Frankenhuyzen J, Vallery H. Design and evaluation of a balance assistance control moment gyroscope. Journal of Mechanisms and Robotics. 2017 Oct 1;9(5):051007.
15. Lemus, D., Berry, A., Jabeen, S., Jayaraman, C., Hohl, K., van der Helm, F. C., ... & Vallery, H. (2020). Controller synthesis and clinical exploration of wearable gyroscopic actuators to support human balance. *Scientific reports*, *10*(1), 10412.
16. Holewijn M. Physiological strain due to load carrying. European journal of applied physiology and occupational physiology. 1990;61:237–245.
17. Schiffman JM, Bensel CK, Hasselquist L, Gregorczyk KN, Piscitelle L. Effects of carried weight on random motion and traditional measures of postural sway. Applied Ergonomics. 2006;37(5):607–614.
18. Heller MF, Challis JH, Sharkey NA. Changes in postural sway as a consequence of wearing a military backpack. Gait & posture. 2009;30(1):115–117.
19. Mohseni O, Berry A, Schumacher C, Seyfarth A, Vallery H, Sharbafi MA. Mediolateral Upper-body Gyroscopic Moment Perturbations During Walking: Exploring Muscle Activation Patterns and Control Strategies. 2024;doi:10.21203/rs.3.rs-3881620/v1.
20. Preece SJ, Alghamdi W. The effect of increasing trunk flexion during normal walking. Gait & Posture. 2021;83:250–255.
21. Sterke BT, Poggensee KL, Ribbers GM, Lemus D, Vallery H. Light-weight wearable gyroscopic actuators can modulate balance performance and gait characteristics: A proof-of-concept study. In: Healthcare. vol. 11. MDPI; 2023. p. 2841.
22. Meijneke C, Sterke B, Hermans G, Gregoor W, Vallery H, Lemus D. Design and evaluation of pint-sized gyroscopic actuators. In: 2021 IEEE/ASME International Conference on Advanced Intelligent Mechatronics (AIM). IEEE; 2021. p. 454–461.

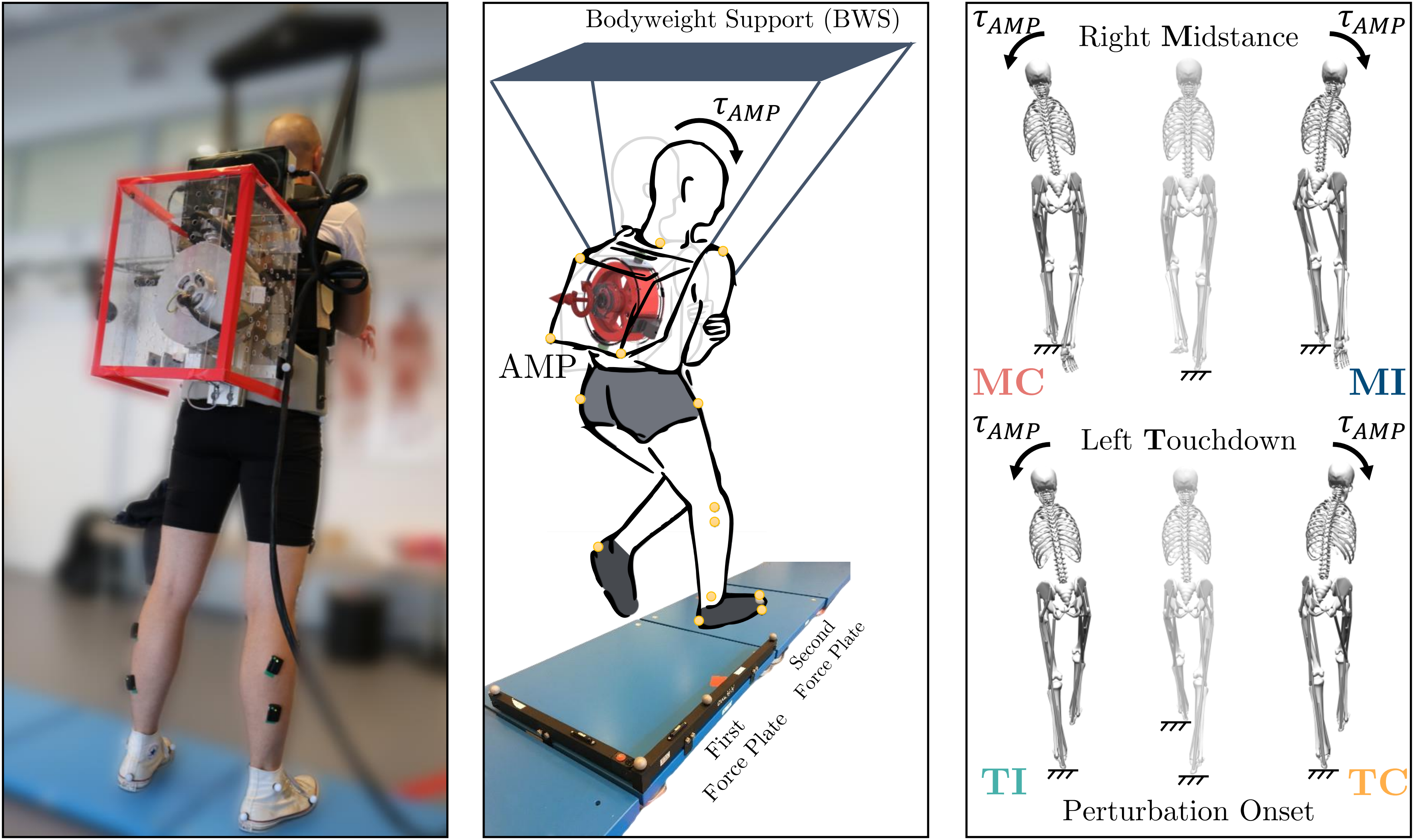
Bodyweight Support (BWS)
τ_{AMP}
AMP
First Force Plate
Second Force Plate
τ_{AMP}
Right Midstance
τ_{AMP}
MC
MI
τ_{AMP}
Left Touchdown
τ_{AMP}
TI
TC
Perturbation Onset

(a) **(b)** **(c)**

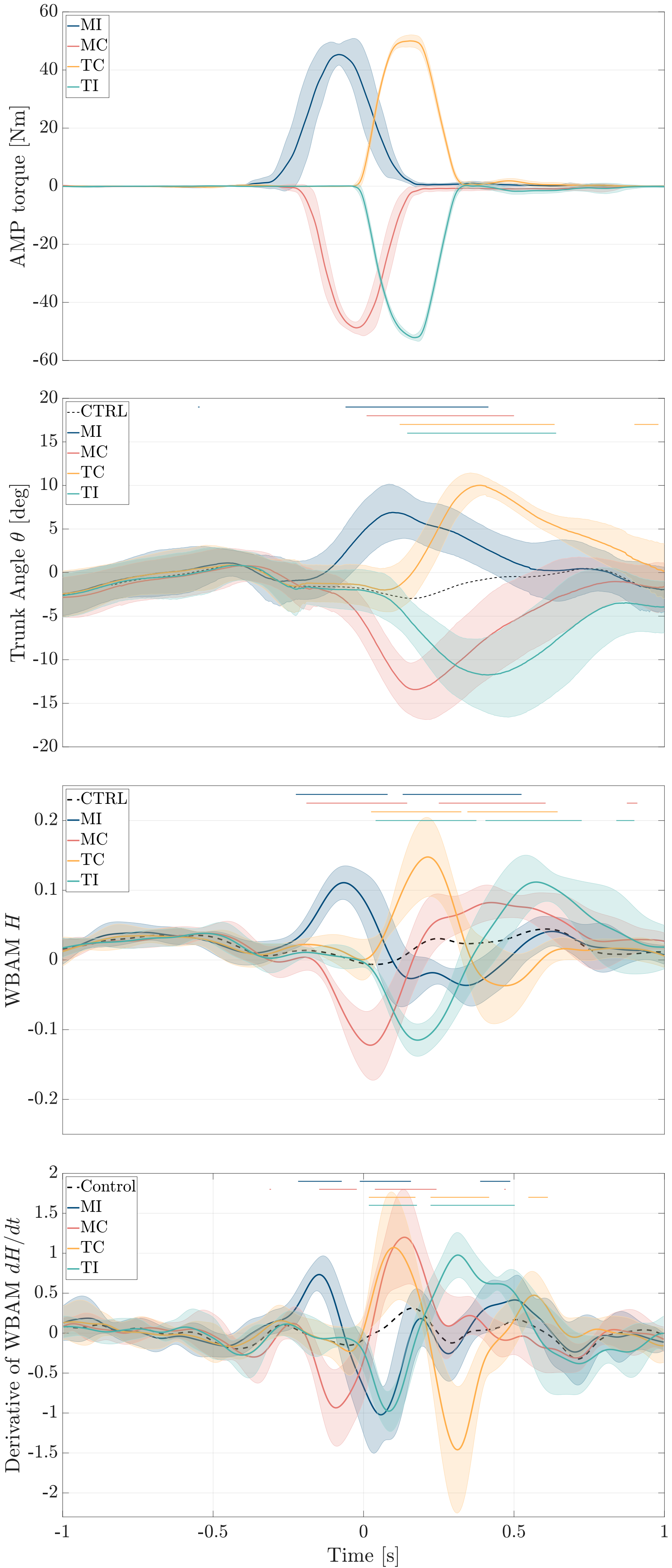

AMP torque [Nm]
60
40
20
0
-20
-40
-60
MI
MC
TC
TI
Trunk Angle θ [deg]
20
15
10
5
0
-5
-10
-15
-20
CTRL
MI
MC
TC
TI
WBAM H
0.2
0.1
0
-0.1
-0.2
CTRL
MI
MC
TC
TI
Derivative of WBAM dH/dt
2
1.5
1
0.5
0
-0.5
-1
-1.5
-2
Control
MI
MC
TC
TI
-1
-0.5
0
0.5
1
Time [s]

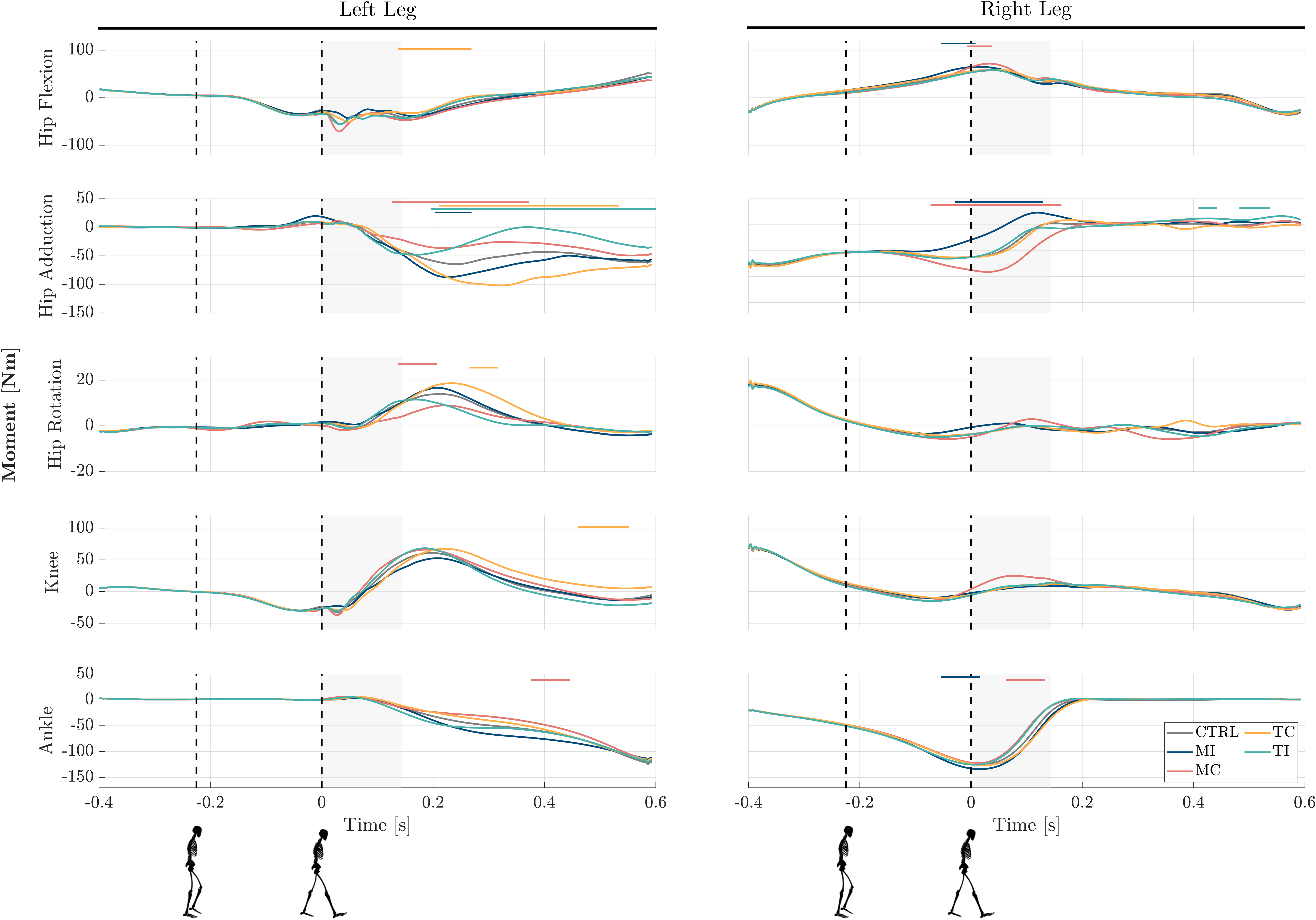

Left Leg
Right Leg
Moment [Nm]
Hip Flexion
Hip Adduction
Hip Rotation
Knee
Ankle
Time [s]
Time [s]
CTRL
TC
MI
TI
MC

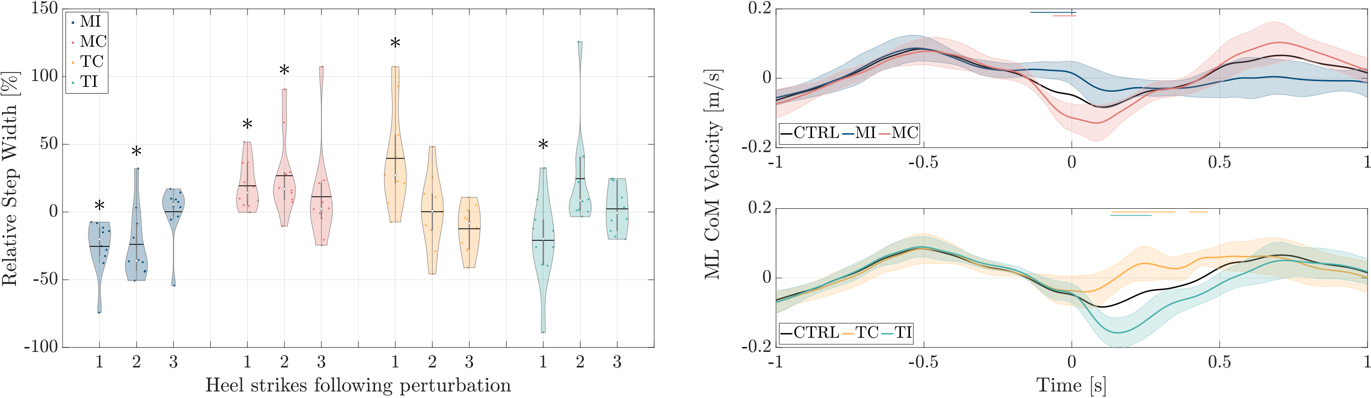

MI
MC
TC
TI
Relative Step Width [%]
Heel strikes following perturbation
ML CoM Velocity [m/s]
CTRL MI MC
CTRL TC TI
Time [s]
